# Detection of *Salmonella* by Surface Plasmon Resonance

**Published:** 2007-08-07

**Authors:** Benjamin Barlen, Saikat Datta Mazumdar, Olga Lezrich, Peter Kämpfer, Michael Keusgen

**Affiliations:** 1 Institut für Pharmazeutische Chemie, Philipps-Universität Marburg, Marbacher Weg 6, D-35032 Marburg, Germany; E-mails: benjamin.barlen@staff.uni-marburg.de; dattamaz@staff.uni-marburg.de; lezrich@web.de; keusgen@staff.uni-marburg.de; 2 Institut für Angewandte Mikrobiologie, Justus-Liebig-Universität Gieβen, Heinrich-Buff-Ring 26-32, D-35392 Gieβen, Germany; E-mail: peter.kaempfer@agrar.uni-giessen.de

**Keywords:** *Salmonella* serovars, lipopolysaccharide (LPS), O-specific antibody, sequential detection, Plasmonic^®^ surface plasmon resonance (SPR) device

## Abstract

This study explores the possibility of simultaneous and specific detection of *Salmonella* serovars by surface plasmon resonance (SPR). The Plasmonic^®^ SPR device was used to develop this rapid assay. The sandwich immunoassay involves the use of a polyclonal anti-*Salmonella* antibody to simultaneous capture multiple *Salmonella* serovars present in a sample. This is followed by specific detection of the captured serovars using O-specific anti-*Salmonella* antibodies. Milk spiked with *Salmonella* Typhimurium and *Salmonella* Enteritidis was used as a model system to establish the assay. The assay was further extended to sequentially differentiate between the two *Salmonella* serovars on a single SPR chip in a single channel. The assay was proved to work without any additional dilution or clean-up steps. The sample volume requirement for the assay is only 10 μL. The lower limits of detection for *Salmonella* Typhimurium and *Salmonella* Enteritidis were 2.50×10^5^ cells mL^−1^ and 2.50×10^8^ cells mL^−1^, respectively.

## 1. Introduction

*Salmonella* serotypes are among the most common bacteria responsible for foodborne gastroenteritis. In the United States alone, approximately 1.4 million human illnesses are reported annually due to salmonellosis caused by *Salmonella* serotypes [1]. The Robert Koch Institute in Germany reported 10,659 cases of salmonellosis during the first four months of 2007 [2]. Recently in Germany (May, 2007), a batch of contaminated dessert resulted in a salmonellosis outbreak causing at least 239 sick and one death [2]. Given the widespread prevalence of *Salmonella* and the consequent threat of salmonellosis, rapid detection of the presence of *Salmonella* in water and foods is of great concern to the food industry [3], the public, and the regulatory agencies [4]. According to the World Health Organisation (WHO), more than 2,500 serotypes of *Salmonella* have been identified till date. Out of these serovars, *Salmonella enterica* serotype Typhimurium (*Salmonella* Typhimurium) and *Salmonella enterica* serotype Enteritidis (*Salmonella* Enteritidis) are the main serovars responsible for foodborne gastroenteritis [1,5]. Studies on trends of the serotypes and host-related factors are necessary for the development of effective prevention plans for salmonellosis. The control of these outbreaks involves the rapid detection of the responsible *Salmonella* serotype.

Different methods have been developed and are used for the detection of *Salmonella* spp. Conventional culture methods for detection of *Salmonella* in foods involve blending of the food product in a non-selective medium to increase the population of the target organism, followed by plating onto selective or differential agar plates to isolate pure cultures [6], and then examining the cultures by phenotypic analysis or metabolic markers. A major drawback is that these methods are labour-intensive, take 2–3 days for results and up to 7–10 days for confirmation [7]. Enzyme-linked immunosorbent assays (ELISA), though faster than the conventional culturing methods, still take up to 3 h and also require labelling reagents [8]. Although recently more rapid and specific immunological assays and methods based on nucleic acid probes and polymerase chain reaction (PCR) have been used, the total time frame is still several hours and requires trained personnel [9,10]. In recent years, there has been a shift in focus to develop biosensors for the rapid detection of pathogens. Surface plasmon resonance (SPR), which belongs to the category of optical biosensors, has been successfully used for the rapid detection of different pathogens [11]. Using SPR technology, it is possible to detect binding events to antibodies without additional labelling steps [12]. The SPR-based assays, besides having the advantages of being label-free and in real-time, are also less time consuming [13].

SPR-based immunoassays for detection of bacteria, including *Salmonella* cells, have been described in literature [14-25]. Most of these assays involve either direct detection of bacteria using polyclonal antibodies or capture and detection of only one single bacterial strain using either polyclonal or monoclonal antibodies. The only literature reference available, for the individual detection of *Salmonella* serovars, uses monoclonal capture antibodies followed by signal enhancement using a polyclonal antibody in different channels of a flow-through SPR system [19]. To our knowledge, there is no literature available on the simultaneous capture of *Salmonella* serovars and specific identification of such captured serovars using SPR. Development of such an assay is important for further enhancing the speed of detection and identification of *Salmonella* serovars in case of outbreaks of salmonellosis.

In this study we report a cuvette-based SPR assay for the specific detection of *Salmonella* serovars using milk as a model food system. Our results show that it is indeed possible to simultaneously capture and distinguish between different serovars of *Salmonella* using SPR either in the multi-channel or in the single-channel sequential detection mode.

## 2. Results and Discussion

### 2.1. Specific detection of Salmonella serovars in buffer

The work presented here is an attempt to establish an SPR-based biosensor for rapid, specific and simultaneous detection of different serovars of *Salmonella* when present singly or as a mixture in one single sample. Initially, the assay was evaluated in phosphate buffered saline (PBS). *Salmonella* Typhimurium and *Salmonella* Enteritidis were each evaluated separately. In the first step, addition of the polyclonal antibody onto the hydrophobic C18 SPR chip resulted in an average detection signal of 60 ± 1.25 AU. The subsequent washing step with PBS did not result in any change in the detection signal, indicating a stable binding of the polyclonal antibody to the SPR chip surface. Blocking of any free available gold surface with bovine serum albumin (BSA), after the immobilisation of the polyclonal antibody, did not result in any significant increase in the SPR detection signal. This was a clear indication of a uniform coverage of the hydrophobic C18 gold surface with the polyclonal antibody. The next step involved the capture of the bacteria using the immobilised polyclonal antibody followed by detection with O-specific antibodies O:4 and O:9 against *Salmonella* Typhimurium and *Salmonella* Enteritidis, respectively.

**Figure 1.**
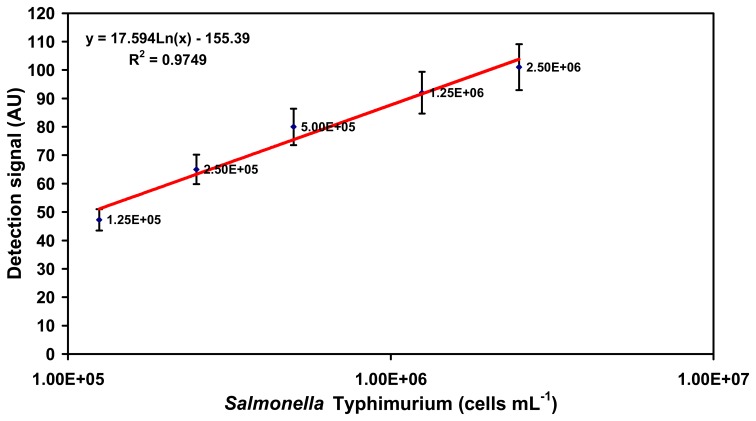
Detection range of SPR-based assay for specific detection of *Salmonella* Typhimurium in PBS buffer system using O-specific O:4 detection antibody. Note that the plot is semi-logarithmic with the cell concentrations increasing exponentially on the X-axis. The signals represented here are those obtained after addition of the O:4 detection antibody.

Different concentrations of both the *Salmonella* serovars were evaluated separately using the assay in PBS. *Salmonella* Typhimurium was found to have a lower limit of detection (LLD, defined as the concentration of cells resulting in a detection signal, which is the average value of the detection signal obtained due to control plus 3 times the standard deviation) of 1.25×10^5^ cells mL^−1^ (47 ± 3.9 AU) when probed with the O:4 detection antibody. The highest concentration of *Salmonella* Typhimurium in PBS evaluated was 2.5×10^6^ cells mL^−1^ resulting in a detection signal of 101 ± 8.3 AU (Fig. 1).

**Figure 2.**
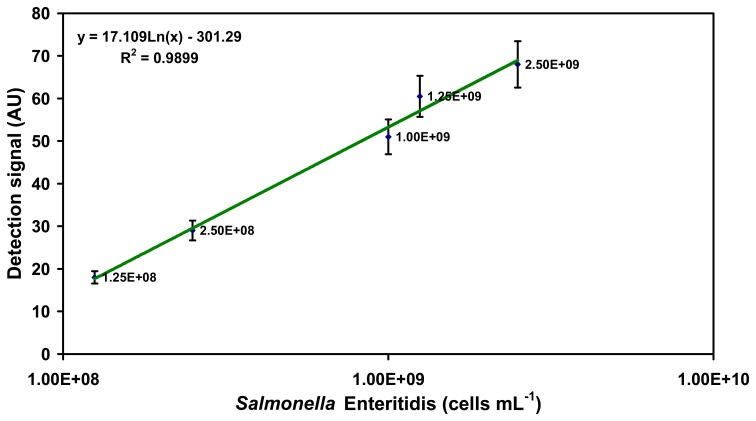
Detection range of SPR-based assay for specific detection of *Salmonella* Enteritidis in PBS buffer system using O-specific O:9 detection antibody. Note that the plot is semi-logarithmic with the cell concentrations increasing exponentially on the X-axis. The signals represented here are those obtained after addition of the O:9 detection antibody.

In case of *Salmonella* Enteritidis the LLD of the assay using the O:9 detection antibody was much higher compared to that of the *Salmonella* Typhimurium. In this case the LLD was 2.5×10^8^ cells mL^-1^ corresponding to a detection signal of 29 ± 4.3 AU. The signal obtained from the highest concentration (2.5×10^9^ cells mL^−1^) of *Salmonella* Enteritidis was 68 ± 5.4 AU (Fig. 2).

The variability in detection limits between the two serovars can be attributed to the difference in affinity of the two detection antibodies towards the respective bacteria. Reports of differences in affinity of different anti-*Salmonella* antibodies against the O-antigens of *Salmonella* are available in literature [26-28]. The possible reason for this difference in affinity is probably the difference in LPS structure of the two bacteria. There is also literature available showing the existence of microheterogeneity in the LPS O-chain of the *Salmonella* serovars Enteritidis and Typhimurium [29].

### 2.2. Specific detection of Salmonella serovars in milk

#### 2.2.1. Detection of each serovar in milk using O-specific antibody

**Figure 3.**
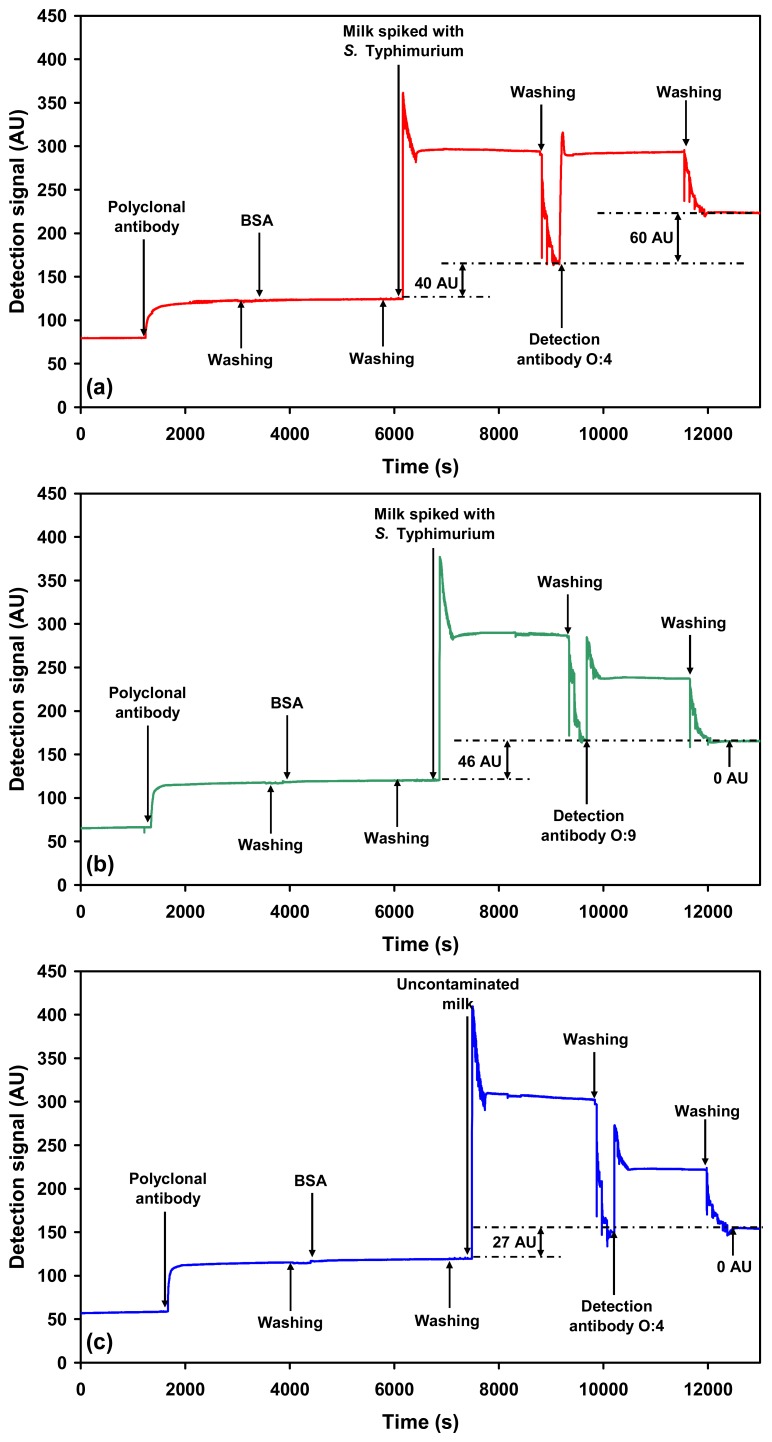
Sensograms showing: (a) Specific detection of *Salmonella* Typhimurium (5×10^5^ cells mL^−1^) in spiked milk using antibody (O:4 detection antibody) against the O:4 antigen after capture from milk using the immobilised polyclonal anti-*Salmonella* antibody. (b) Cross-reactivity check using spiked milk containing *Salmonella* Typhimurium (5×10^5^ cells mL^−1^) against O:9 detection antibody, which is specific for *Salmonella* Enteritidis. (c) SPR response to probing of uncontaminated milk (control) using the *Salmonella* Typhimurium specific O:4 detection antibody.

**Figure 4.**
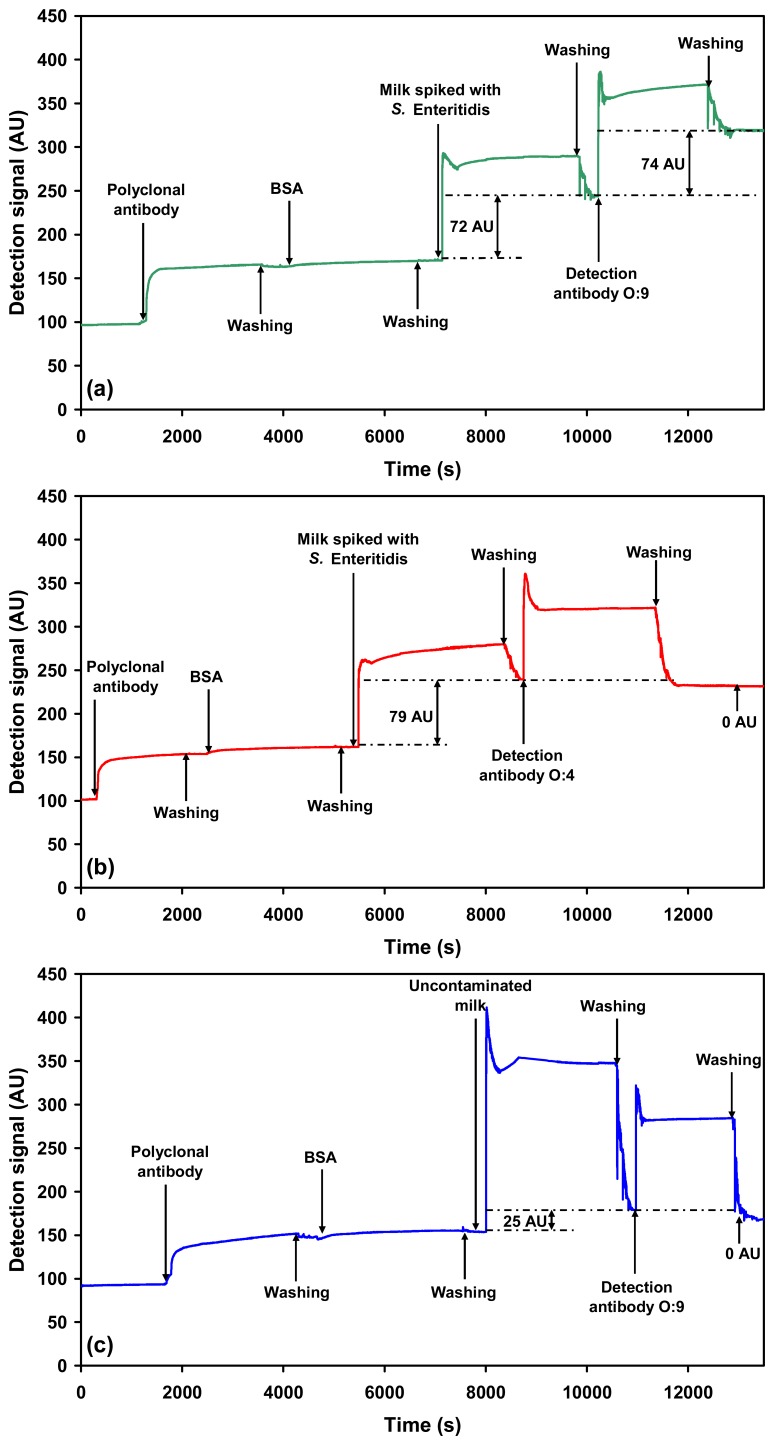
Sensograms showing: (a) Specific detection of *Salmonella* Enteritidis (3×10^9^ cells mL^−1^) in spiked milk using antibody (O:9 detection antibody) against the O:9 antigen after capture from milk using the immobilised polyclonal anti-*Salmonella* antibody. (b) Cross-reactivity check using spiked milk containing *Salmonella* Enteritidis (3×10^9^ cells mL^−1^) against O:4 detection antibody, which is specific for *Salmonella* Typhimurium. (c) SPR response to probing of uncontaminated milk (control) using the *Salmonella* Enteritidis specific O:9 detection antibody.

To test how the assay performs in a complex food matrix, milk spiked with the *Salmonella* serovars was used as a model system. Addition of milk spiked with *Salmonella* Typhimurium or *Salmonella* Enteritidis onto the sensor chip coated with polyclonal antibody resulted in an initial significant increase in the detection signal. This detection signal, however, was reduced after the subsequent washing step with PBS. The initial increase is attributed to bulk refractive index change of the sample medium due to the milk matrix. The detection signal due to capture of *Salmonella* Typhimurium (5×10^5^ cells mL^−1^) using the polyclonal capture antibody was only 43 ± 4.5 AU (Fig. 3a). The corresponding detection signal due to the captured *Salmonella* Enteritidis (3×10^9^ cells mL^−1^) from spiked milk using the polyclonal capture antibody was 75 ± 5.0 AU (Fig. 4a).

The captured *Salmonella* Typhimurium or *Salmonella* Enteritidis was then probed with the respective O-specific detection antibodies. The final detection signal obtained for the highest concentration of *Salmonella* Typhimurium (5×10^5^ cells mL^−1^) probed using the O:4 detection antibody was 56 ± 3.6 AU (Fig. 3a). The LLD of *Salmonella* Typhimurium in milk using O:4 detection antibody was 2.5×10^5^ cells mL^−1^. In both cases the signal due to control (uncontaminated milk) was 0 AU (Figs. 3c and 4c).

In case of *Salmonella* Enteritidis the highest concentration probed using the O:9 detection antibody was 3×10^9^ cells mL^−1^ resulting in a detection signal of 68 ± 5.4 AU (Fig. 4a). The LLD of *Salmonella* Enteritidis in milk, using O:9 detection antibody, was 2.5×10^8^ cells mL^−1^. It is important to note here, that even though the LLD in case of *Salmonella* Enteritidis is higher as compared to that of *Salmonella* Typhimurium, the LLD of both the serovars are similar in buffer and in milk. This observation is also valid for the detection of *Salmonella* using a polyclonal detection antibody, recently published by us [14]. Thus, there is no compromise on the overall detection capability of the assay due to the milk matrix. The possible reasons for the difference in detection limits between the two serovars are already discussed above.

**Figure 5.**
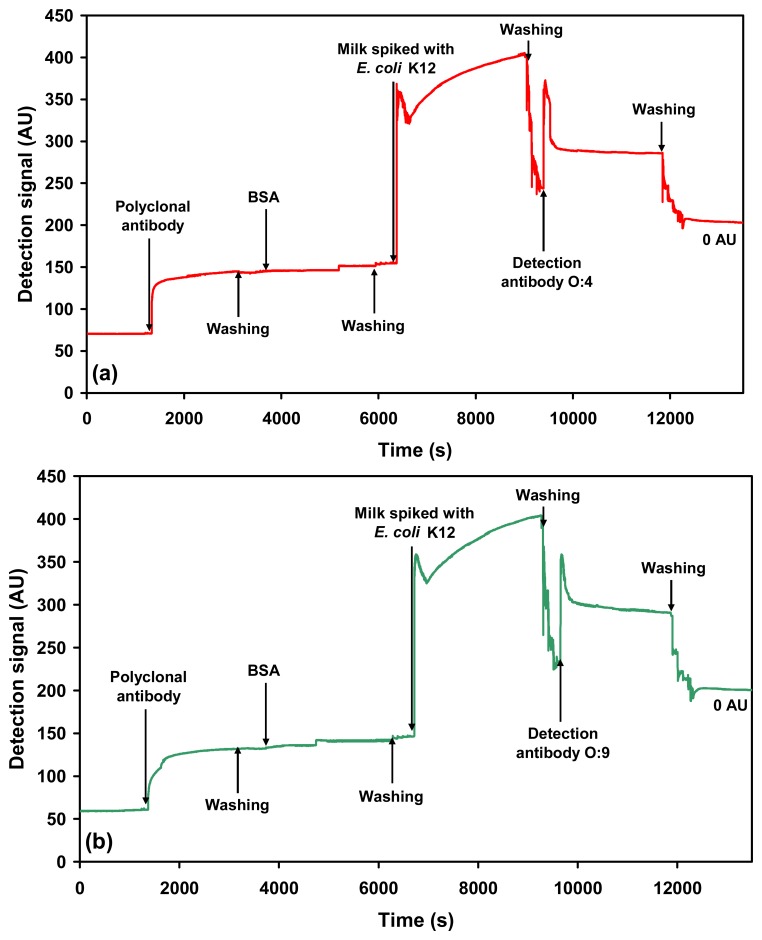
Sensograms showing: (a) Cross-reactivity check using spiked milk containing *E. coli* K12 (1.0×10^9^ cells mL^−1^) against O:4 detection antibody, which is specific for *Salmonella* Typhimurium. (b) Cross-reactivity check using spiked milk containing *E. coli* K12 (1.0×10^9^ cells mL^−1^) against O:9 detection antibody, which is specific for *Salmonella* Enteritidis. Both antibodies show no cross-reactivity to *E. coli* K12 (0 AU).

Cross-reactivity of the O:4 detection antibody to *Salmonella* Enteritidis (Fig. 4b) and O:9 detection antibody to *Salmonella* Typhimurium was also evaluated (Fig. 3b). As expected, the antibodies were found to be specific for the respective serovars. Furthermore, no cross-reactivity was observed when milk spiked with *E. coli* K12 (1.0×10^9^ cells mL^−1^) was probed using the O-specific antibodies (Fig. 5).

#### 2.2.2. Individual detection of serovars in a mixture using monoclonal O-specific antibodies

The next step of the assay development was to detect both the *Salmonella* serovars when present together in a mixture. Initial experiments were carried out to understand the mode of interaction of the serovars and the antibodies to each other. Milk was spiked with a mixture of *Salmonella* Typhimurium and *Salmonella* Enteritidis having the same final concentration of each bacterium as when tested singly. In other words, the highest concentration of the tested samples were 5×10^5^ cells mL^−1^ and 3×10^9^ cells mL^−1^ of *Salmonella* Typhimurium and *Salmonella* Enteritidis, respectively. The control milk samples were prepared by making the necessary volume corrections with respect to the samples spiked with bacteria.

**Figure 6.**
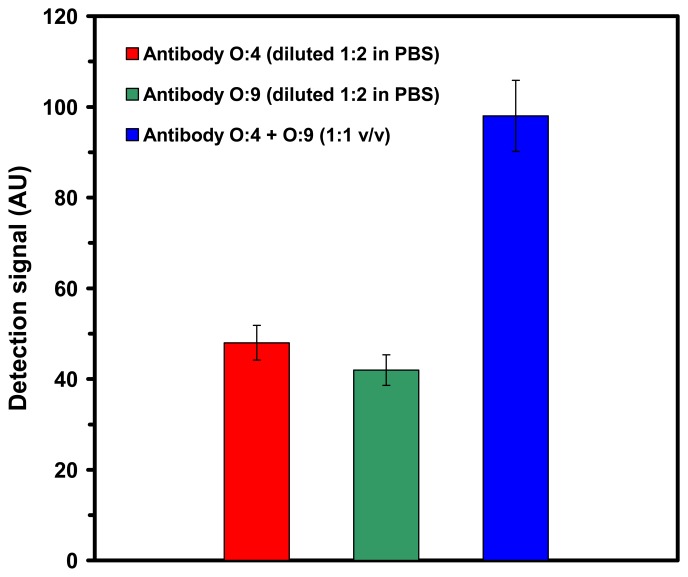
Detection signals of the SPR assay for the specific detection of *Salmonella* serovars in milk samples spiked with a mixture of *Salmonella* Typhimurium and *Salmonella* Enteritidis, using O-specific detection antibodies. The signal due to control (uncontaminated milk) was 0 AU.

The mixture was initially probed with either O:4 detection antibody or O:9 detection antibody (both diluted 1:2 in PBS) and resulted in an average detection signal of 45 ± 4.2 AU. When the same mixture of serovars in milk was probed with a mixture of the undiluted O:4 and O:9 antibodies (1:1 v/v) the resulting detection signal (98 ± 7.8 AU) obtained was nearly an addition of the individual detection signals of the respective antibodies (Fig. 6).

These results clearly indicate that the serovars, when present in a mixture, do not interfere with their interactions with the respective O-specific detection antibodies. The results support the possibility of developing assays for the simultaneous detection of serovars.

Based on the above results, further experiments were carried out to distinguish between the two *Salmonella* serovars when present together in milk. After simultaneous capture of the two serovars from the spiked milk sample by the polyclonal capture antibody, detection was carried out using the undiluted O-specific antibodies. This step required the use of different channels (multi-channel detection) for the O:4 and the O:9 detection antibodies, respectively. In this case, the results obtained for probing the mixture of serovars with the undiluted detection antibodies were similar to that for the detection of each individual serovar in spiked milk.

#### 2.2.3. Sequential detection of serovars in a mixture

**Figure 7.**
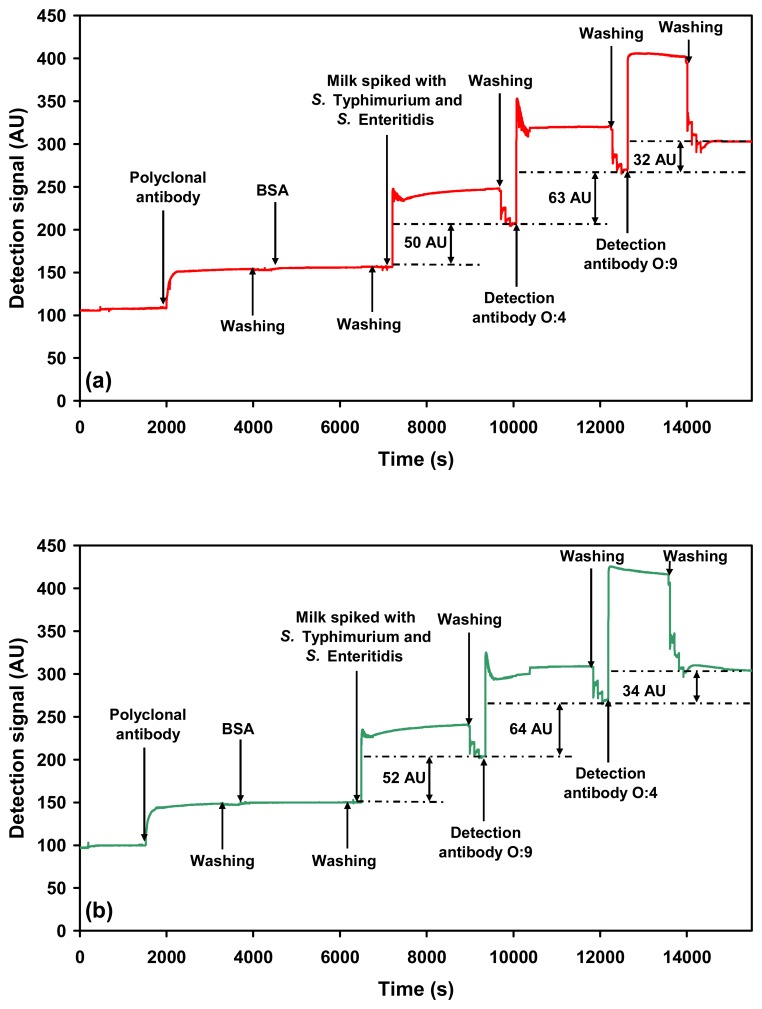
SPR sensograms showing sequential detection of *Salmonella* serovars spiked in milk: (a) *Salmonella* Typhimurium (O:4 detection antibody) followed by *Salmonella* Enteritidis (O:9 detection antibody). (b) *Salmonella* Enteritidis (O:9 detection antibody) followed by *Salmonella* Typhimurium (O:4 detection antibody).

As it was already established that neither the serovars nor the detection antibodies interfere with the SPR detection process when present together in a mixture, further experiments were carried out to evaluate the possibility of detecting both the serovars, using a single SPR channel in a sequential manner. In the sequential detection mode, the addition of the milk sample containing the mixture of both the *Salmonella* serovars (5×10^5^ cells mL^−1^ of *Salmonella* Typhimurium and 3×10^9^ cells mL^−1^ of *Salmonella* Enteritidis) onto the sensor chip was then probed with either O:4 or O:9 detection antibody, followed by O:9 or O:4 detection antibody. The first detection signals were comparable to that obtained in the multi-channel detection mode. The average value of the detection signal for *Salmonella* Typhimurium in the mixture when probed with O:4 detection antibody first was 66 ± 3.2 AU (Fig. 7a). The corresponding detection signal for *Salmonella* Enteritidis in the mixture when O:9 detection antibody was the first antibody was 60 ± 6.7 AU (Fig. 7b). The detection signal for *Salmonella* Typhimurium when detected in the second place (O:4 detection antibody) was 40 ±7.8 AU (Fig. 7b), and the corresponding detection signal for *Salmonella* Enteritidis detected secondly (O:9 detection antibody) was 28 ± 5.7 AU (Fig. 7a).

The data clearly indicate a reduction in detection signal when either of the detection antibodies is added in the second place. In comparison, the detection signal obtained when either of the antibodies is added in the first place is always higher in the sequential detection mode. This reduction in detection signal is explained as follows:

In case of SPR an evanescent field is generated at the metal/dielectric interface by the surface plasmon wave. The unique characteristic of the evanescent field is, that the field amplitude is greatest at the interface and exponentially decays as a function of distance from the metal/dielectric interface [30]. For biomolecular interaction studies using SPR, the evanescent field intensity is effective only up to a depth of 100-200 nm [13].

*Salmonellae* belong to the family Enterobacteriaceae and are typically 1-5 μm in diameter [31]. Hence, the size of the bacteria places the bulk of the bound cells outside the SPR evanescent field, much beyond the effective penetration depth of 100-200 nm [13]. This reason for high detection limits for bacterial detection using SPR have been documented in literature [21]. Consequently, in the sequential detection mode further addition steps beyond a particular point would result in a lowering of the detection signal, due to the presence of analytes outside the effective evanescent field. This explains the reduction in signal obtained for the second detection antibody in the sequential detection mode.

**Figure 8.**
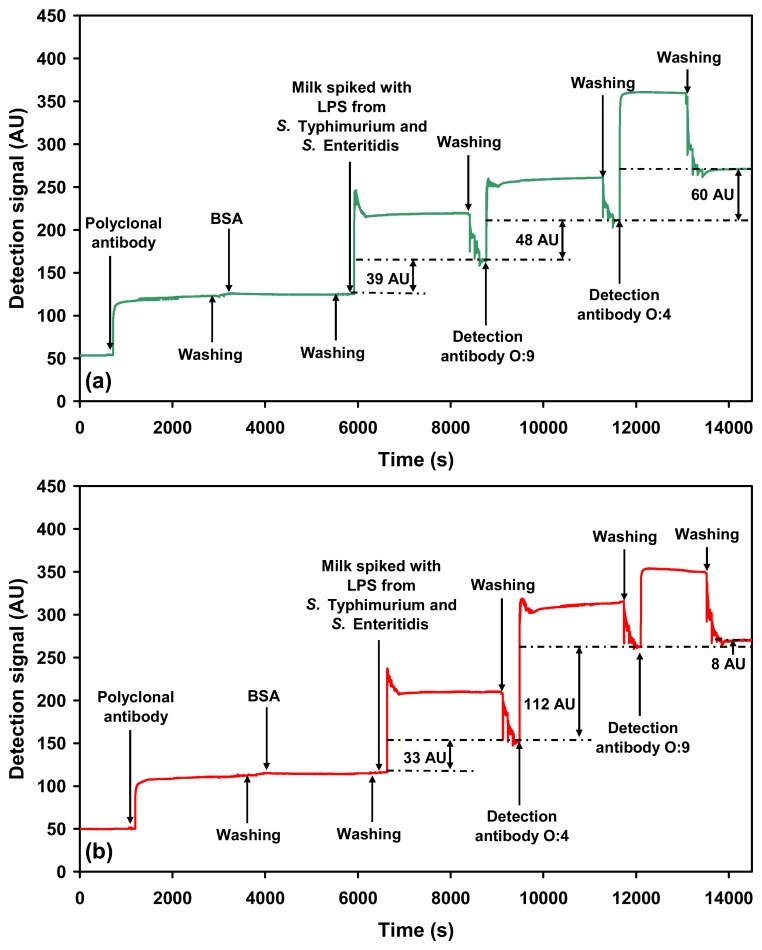
SPR sensograms showing sequential detection of a mixture of LPSs from *Salmonella* serovars spiked in milk: (a) *Salmonella* Typhimurium LPS (O:4 detection antibody) followed by *Salmonella* Enteritidis LPS (O:9 detection antibody). (b) *Salmonella* Enteritidis LPS (O:9 detection antibody) followed by *Salmonella* Typhimurium LPS (O:4 detection antibody).

Studies were further carried out to elucidate and understand the phenomenon of sequential detection using SPR with respect to the detection of gram-negative bacteria. In order to focus only on the bacterial component responsible for the SPR signal in the assay, purified LPS of both the *Salmonella* serovars were further tested using the SPR assay. Milk was spiked with equal concentrations of both the LPSs (40 μg mL^−1^ each). The spiked sample was then probed in the sequential detection mode; O:4 followed by O:9 and also O:9 followed by O:4. In both cases, controls were run using uncontaminated milk with the relevant dilution corrections. The data clearly show that the O:9/O:4 detection mode was able to differentiate between both the LPSs (Fig. 8a). The SPR detection signals were 47 ± 2.1 AU for O:9 as the first and 63 ± 6.1 AU for O:4 as the second detection antibody. In case of the O:4/O:9 mode of detection the signals obtained were 112 ±8.9 AU and 8 ±1 AU, respectively (Fig. 8b).

It is evident from the data that between both the LPSs, the LPS from *Salmonella* Enteritidis has a lower detection signal in comparison to that of *Salmonella* Typhimurium LPS, even though both are present at the same concentration. This finding agrees well with the observations already made here using whole bacterial cells of both the serovars. It is thus clear that the signal due to *Salmonella* Enteritidis LPS would be lost if detected in the second place of the sequential detection mode.

Hence, using the sequential detection mode, it is always an advantage to have a prior knowledge about the range of detection signals of both the bacteria on SPR when detected singly. As there is early signal saturation and a consequent decrease in the second signal in the sequential detection mode, it is appropriate that the serovar having the lower detection signal is detected in the first place. These observations are in agreement with the only available published data, for the sequential detection of a mixture of anti-BSA antibodies and horseradish peroxide using SPR [32].

Based on this study, using whole cells and purified LPSs of the respective serovars, it can be concluded that sequential detection of serovars using SPR can be easily achieved. However, as in case of all assays, a few optimisation steps in terms of determining the order of detection of the serovars in the sequential detection mode need to be carried out. This would involve initial screening of the individual serovars using SPR in the multi-channel mode to determine their SPR detection signals and detection limits. Using this data, single-channel sequential detection can then be designed. The serovar with the lower detection signal in the individual detection mode is detected first, followed by the other serovar of interest. Such an assay would further increase the capability of SPR assays to quickly screen a number of serovars and bacterial strains, reducing time and costs of the assay.

Though, the above work has been able to establish an SPR assay for specific detection of *Salmonella* serovars and also a scheme for sequential detection of the bacteria, there is further scope of improving the assay in terms of detection limits. A look at other recent publications in the area of bacterial biosensing clearly brings out this point. The detection limit for an array-based biosensor is reported to be 5×10^3^ cells mL^−1^ for the detection of *Escherichia coli* O157:H7 [33]. The detection limit for *Salmonella* detection with a direct-binding optical grating coupler (OGC) immunosensor is reported to be 1.3×10^3^ CFU mL^−1^[34]. Assays developed using leaky waveguide sensor devices (LWD) have reported detection limits of 1×10^3^-1×10^4^ spores mL^− 1^ for detection of bacteria using bacterial spores [35]. Attempts to improve detection limits of SPR-based assays for bacterial detection have been successful, e.g. use of protein G as a spacer molecule on the gold surface to orient the immobilised capture antibodies. This particular assay, using protein G, was able to detect *Salmonella* Typhimurium down to 1×10^2^ cells mL^−1^ in buffer [20]. The use of gold nanoparticles to improve the sensitivity of SPR assays is also possible [36]. Our future work will thus focus on further improving the detection limits of the present assay.

In conclusion, the possibility of using SPR to detect and differentiate *Salmonella* serovars when present together in a given sample has been demonstrated using milk spiked with a mixture of *Salmonella* serovars Typhimurium and Enteritidis. The limits of detection of the assay are 2.5×10^5^ cells mL^−1^ for *Salmonella* Typhimurium and 2.5×10^8^ cells mL^−1^ in case of *Salmonella* Enteritidis. The assay involves simultaneous capture of the *Salmonella* serovars using a polyclonal anti-*Salmonella* antibody. This is followed by specific detection of the captured serovars using O-specific antibodies against each serovar.

The study also explored the possibility of a sequential detection protocol for the detection of *Salmonella* serovars using a single SPR channel. Using the sequential detection mode, it was possible to differentiate between *Salmonella* Typhimurium and *Salmonella* Enteritidis when present together as a mixture in milk. The sequential assay was proved to work for both whole cells and purified lipopolysaccharide (LPS). A general rule for sequential detection of two bacterial mixtures using SPR has also been established. It was shown that in case of the sequential detection mode the bacteria having a lower SPR response to the detection antibody should be detected first. This is because sequential detection results in a decrease in signal of the second detection antibody used for detection of the second serovar. In the multi-channel detection mode the time required for analysis of each serovar is only 1 h, with the added advantage of having real-time data of the binding events. The single-channel sequential detection mode would increase the capability to detect more number of samples on a single chip in the same time. The sample requirement for each analysis is only 10 μL.

The results of this work can provide useful leads for further development of SPR-based biosensors as well as other biosensors for multiple and specific detection of *Salmonella* and other bacteria. Besides finding use as a high throughput microbiological safety tool in the food industry, the assay can also be used as a rapid detection tool for identification of *Salmonella* serovars involved in outbreaks of salmonellosis. Thus, the assay has the potential to be a valuable tool in control of salmonellosis.

## 3. Experimental Section

### 3.1. Chemicals

Polyclonal rabbit anti-*Salmonella* spp. antibody (IgG) was obtained from Capricorn Products, Portland, USA. O-specific (O:4 and O:9) antibodies against *Salmonella* Typhimurium and *Salmonella* Enteritidis were obtained from SIFIN (Berlin, Germany). The killed *Salmonella* Typhimurium and *Salmonella* Enteritidis cells used in this assay were obtained from the Rheinische Friedrich-Wilhelms-Universität (Bonn, Germany) and the Justus-Liebig-Universität (Gieβen, Germany). Lipopolysaccharide (LPS) from *Salmonella* Typhimurium and *Salmonella* Enteritidis, prepared by the phenol extraction method, was obtained from Sigma-Aldrich (Germany). The *Escherichia coli* K12 cells used in the cross-reactivity tests were cultured in our laboratory. Luria–Bertani (LB) agar and LB broth, both used for culturing, and thimerosal used as an agent to kill the bacteria were all purchased from Merck (Darmstadt, Germany). Bovine serum albumin (BSA) was purchased from Merck (Darmstadt, Germany). Bronidox^®^L, used as a preservative in buffers, was obtained from Sigma-Aldrich (Germany). Phosphate buffered saline (PBS, 0.15 M, pH 7.3, containing 0.12% Bronidox^®^L) used in all experiments was prepared in our laboratory. Water used was obtained from a PURELAB^®^ Plus unit (USF Elga, Germany). Other chemicals were purchased from standard commercial sources and were of analytical grade. Milk used in these experiments was obtained from the local supermarket (Ultra-high-temperature-treated, homogenized, 1.5% fat) or in case of fresh milk from a local farm.

### 3.2. Preparation of Salmonella antigen

The bacteria *Salmonella enterica* subsp. *enterica* serovar Typhimurium and *Salmonella enterica* subsp. *enterica* serovar Enteritidis were grown in sterile liquid LB medium by incubation for 24 h at 37 °C. Subsequently, in order to kill the bacteria, thimerosal (1%, w/w) was added to the medium and incubated at ambient temperature for 1.5 days. The contents were vortexed at regular intervals during this time. After this period, to check the effectiveness of the thimerosal treatment, 100 μL of the LB medium, containing bacterial cells, was added onto sterile LB agar medium. In case of a successful thimerosal treatment, there should be no visible growth of bacteria on the LB agar after 48 h of incubation at 28 °C. The liquid medium, containing the killed bacteria, was then centrifuged at 4,000 rpm for 10 min at ambient temperature using a 6K10 centrifuge (Sigma, Osterode, Germany). The cells were obtained as pellets at the bottom of the tube. After pouring off the supernatant, the pellets were washed three times with PBS. In each case this was done by suspending the pellets in PBS followed by renewed centrifugation. After the third and final wash, the pellets were suspended in PBS to the initial volume and stored at 4 °C until further use.

### 3.3. Surface coating

The gold surface of each prism was modified to create a hydrophobic surface, henceforth referred to as C18. This method of modification of the gold surface of the SPR prism has been recently reported by our working group [14]. Briefly, the gold prisms were first cleaned in acetone for 10 min, followed by incubation in a mixture of 0.1 M potassium hydroxide and 30% hydrogen peroxide for 20 min. The gold prisms were then rinsed with water, followed by incubation in a solution of C18 alkylsilane for 6 h at room temperature. The C18 gold prisms were finally dried under vacuum and stored until further use.

### 3.4. Surface plasmon resonance device

**Figure 9.**
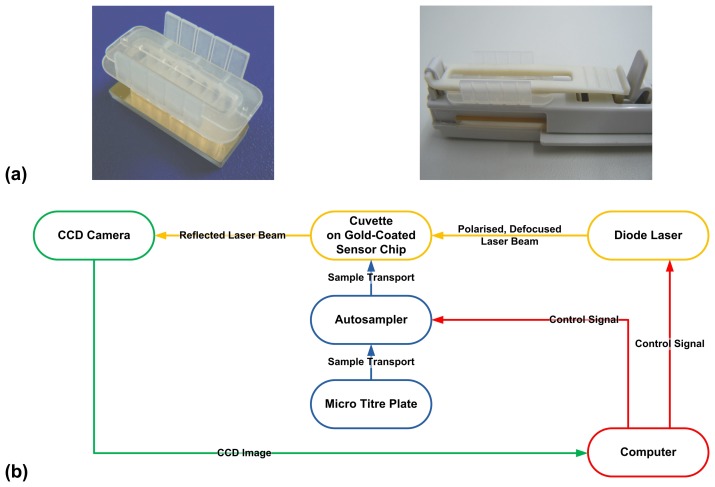
(a) Photographs showing (left) the cuvette chamber, with 8 channels, placed on top of the gold prism (right) the prism with the cuvette chamber mounted on the prism holder. (b) Schematic representation of the setup of the Plasmonic^®^ SPR device.

The assay described here was developed on the Plasmonic^®^ SPR device (Plasmonic Biosensoren AG, Wallenfels, Germany). The device works on the well-known Kretschmann-Raether attenuated total reflection (ATR) configuration [37]. Each SPR chip is made up of a glass prism coated uniformly with gold to a thickness of 50 nm, on the reflecting surface. The device is characterised by a cuvette system. The surface of the gold-coated SPR prism forms the bottom of the cuvette, providing 8 parallel channels (Fig. 9a).

The samples and reagents are loaded into a micro titre plate, from where the autosampler loads them automatically into the channels of the cuvette. The autosampler is controlled by a computer. The advantage of this cuvette-based system, in comparison to fluidic systems, is that the sample materials can be examined without danger of blockage. Furthermore, a sample volume of only 10 μL is required for analysis. The Plasmonic^®^ uses defocussing optics. The source of incident light is a laser diode (786 nm). It emits an elliptical beam of light, which is then converted, using the cylindrical lens system of the device, into a divergent beam. Using the defocussing optics, it is possible to cover all possible angles of incidence required for the real-time determination of the SPR angle, on the gold surface. The reflected light is detected with the help of a charge-coupled device (CCD) camera. The temperature is controlled by means of Peltier elements. The setup of the SPR device is shown schematically above (Fig. 9b).

### 3.5. Measurement conditions

All detection signals obtained for the assays on the SPR device are with reference to the refractive index of PBS on the chip surface. All experiments were carried out at a constant temperature of 22.00 °C.

### 3.6. Assay setup

**Figure 10.**
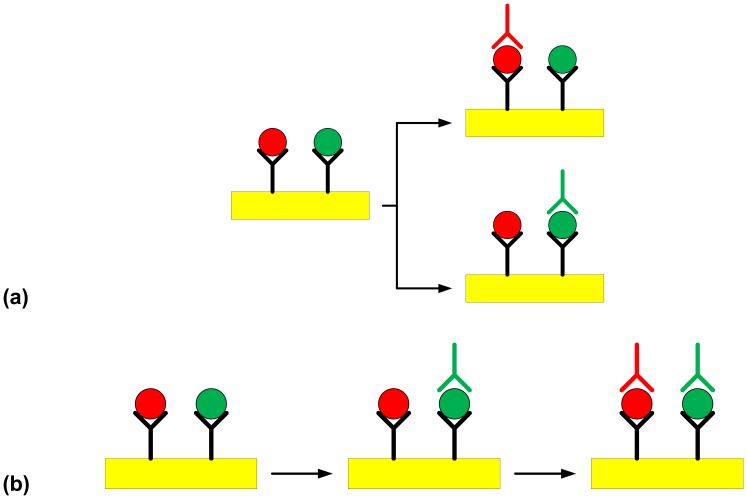
Schematic representation of the two SPR-modes for the detection of *Salmonella* serovars ( 
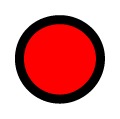
 = *S*. Typhimurium, 
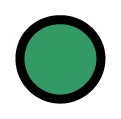
 = *S*. Enteritidis) using the Plasmonic SPR device ( 
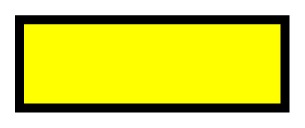
 = C18-modified gold surface, Y = polyclonal capture antibody, 
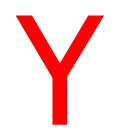
 = O:4-specific detection antibody against *S*. Typhimurium, 
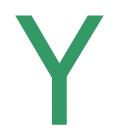
 = O:9-specific detection antibody against *S*. Enteritidis). Steps involved in (a) the multi-channel SPR detection of *Salmonella* serovars present in a sample, (b) the sequential single-channel detection for *Salmonella* serovars present in a sample.

In the first step of the assay, polyclonal antibody (250 μg mL^−1^) against *Salmonella* spp. is immobilised on the SPR chip. The immobilised polyclonal antibodies are then used to capture all *Salmonella* serovars that may be present in a given sample. After the step involving immobilisation of the polyclonal antibody and before capture of bacteria, any unbound polyclonal antibodies were washed away with PBS. Any available free gold surface was then blocked using BSA (100 μg mL^−1^) in order to prevent any non-specific binding of the bacteria. The captured bacteria were then further probed with monoclonal antibodies specific for each serovar of interest. The binding of the bacterial cells to the immobilised capture antibody was recorded in real-time on a SPR sensogram, in terms of arbitrary units (AU). Using the Plasmonic^®^ SPR device it was possible to explore two different modes of specific detection after the first “polyclonal capture” step. Each serovar was probed with O-specific detection antibodies, either in different channels (multi-channel detection) of the device (Fig. 10a), or a sequential detection mode was explored using only one channel (single-channel detection), for detection of both the captured serovars (Fig. 10b).

First, the assay was carried out in a buffer system in the multi-channel detection mode, followed by validation in milk. The bacteria, *Salmonella* Typhimurium and *Salmonella* Enteritidis, were first captured, using the immobilised polyclonal antibody. Individual serovars were probed in buffer and also in spiked milk using the respective O-specific antibodies. This was followed by detection of the serovars in samples having a mixture of both serovars. Cross-reactivity of the O-specific detection antibodies against each of the two serovars was also tested in the multi-channel detection mode.

The effect of addition of a mixture of both the detection antibodies to a mixture of both the serovars was also compared with that of individual detection of each serovar in the mixture using each O-specific antibody. After validation of the multi-channel detection mode a sequential detection mode was then further explored using the same *Salmonella*-spiked milk samples. In the sequential detection mode, the *Salmonella* serovars captured on the SPR chip from a mixture in milk were probed with O:4 detection antibody followed by O:9 detection antibody or vice versa.
